# The Effect of Good Enough Sex (GES) Model-Based Sexual Counseling Intervention on the Body Image in Women Surviving Breast Cancer: A Randomized Clinical Trial

**DOI:** 10.31557/APJCP.2021.22.7.2303

**Published:** 2021-07

**Authors:** Farnaz Farnam, Zohreh Khakbazan, Saharnaz Nedjat, Saeed Razavi Dizaji, Samira Barjasteh

**Affiliations:** 1 *Department of Reproductive Health Midwifery, School of Nursing & Midwifery, Tehran University of Medical Sciences, Tehran, Iran. *; 2 *Department of Epidemiology and Biostatistics, School of Public Health, Tehran University of Medical Sciences, Tehran, Iran. *; 3 *Department of Medical Oncology and Hematology, Urmia University of Medical Sciences, Urmia, Iran. *

**Keywords:** Sexual counseling, breast cancer, body image

## Abstract

**Introduction:**

Women undergoing breast cancer treatment experience a number of changes, including loss or deformity of one or both breasts, surgical wounds, skin changes, and weight gain. These changes are very closely related to physical appearance and body image and often lead to reduced mental health, marital quality and psychological stress.

**Objective:**

This study aimed to determine the effect of Good Enough Sex (GES) model-based counseling intervention on the body image in women surviving breast cancer.

**Patients and Methods:**

This randomized clinical trial was a kind of quantitative study with control group carried out on 100 women (50 women in the intervention group and 50 women in the control group) who randomly entered into the study after completing the informed consent form. The intervention included 4 sessions of 120-190 minute sexual counseling with 2 and 3 months follow-up. The data were collected consisting of demographic characteristics and disease-related information, Body Image Scale (BIS) and analyzed using ANOVA repeated measures.

**Results:**

The results showed a statistically significant difference between the mean of body image in the intervention and control groups (P <0.001).

**Conclusion:**

The educational and counseling intervention program based on a socio-psychological model was effective in improving the body image among women surviving breast cancer. The inclusion of educational and counseling programs in service centers in this regard has an effective role in the reproductive health of women with breast cancer.

**Trial Registration:**

Registration of trial protocol has been approved in Iranian registry of clinical trials (IRCT20120609009975N8, https://en.irct.ir/trial/42030, ethical code; IR.TUMS.FNM.REC.1396.4865).

## Introduction

Today, cancer is one of the most important health problems worldwide (Siegel et al., 2015). Among cancers, breast cancer is the most common cancer known in women (DeSantis et al., 2014). Over the past 20 years, the prevalence of breast cancer and its mortality rate in developing countries has increased rapidly(Jemal et al., 2010). Breast cancer accounts for 4.24% of all cancers among Iranian women (Harichi et al., 2011). One eighth of women is likely to develop the cancer in their lifetime(Anderson, 2006). Also, the age of breast cancer in Iran is 10 years younger than developed countries, and 70% of cases are diagnosed in the advanced stage of the disease (Rafiemanesh et al., 2016). Development of cancer can affect various aspects of a person’s life as a stressful event (Sarenmalm et al., 2013). Following changes in the physical and mental level, the patient’s social relationships and intimate interactions with others are transformed and the patient feels disrupted in his social life. Breast cancer impairs sexual function and self-esteem and causes negative body image, decreased femininity and decreased sexual activity (Boehmer et al., 2014). Women who have an unfavorable image of their body suffer from low self-esteem, sexual dysfunction and reduced quality of life. However, the goal of treatment and diagnostic methods in recent decades is not only to relieve the patient’s symptoms and survival, but also to maintain a quality of life as well (Gavric and Vukovic-Kostic, 2016). However, concerns about the impact of invasive breast cancer treatments, including mastectomy, on patients’ lives are increasing (Najafi et al., 2015). and the prevalence of mastectomy as a surgical treatment for breast cancer in Iran, lack of attention to its psychosocial consequences and lack of attention to postoperative rehabilitation of patients doubles the need for repeated studies in the field of design and evaluation of psychosocial and sexual interventions in the field of reproductive health of these patients in Iran. 

Therefore, in this study, for the first time in Iran, the effect of Good Enough Sex (GES) counseling model on the body image of mastectomy women was assessed. GES model intends to create an integrated approach to sexual health and treatment as a simple and constructive treatment plan for couples. Its content is a realistic understanding of sexual value. GES model focuses on individual beliefs and superstitions and unusual expectations of sex that are born of the media in the new age. The irrational expectations that people have about sex and married life are like a mirage that usually has no positive consequences. Sexual and marital counseling is a good opportunity for couples to end their conflicts and contradictions following surgery and treatment in physical appearance, and by learning the skills of playing love with their spouse, proper communication with their spouse, increasing empathy, respect and mutual understanding to get closer to having a positive attitude towards yourself and proper sex (Metz et al., 2007). The concept of GES challenges couples to have positive and realistic expectations about their sexual organs as well as their psychological and interpersonal dimensions. While societies are filled with images and legends of beauty, hyperreal sexual performance and an overemphasis on the romantic and ideal aspects of sex, this model teaches sexual problems occur periodically in people’s lives, sexual pleasure varies throughout life, especially if couples face a crisis such as a conflict with an incurable and chronic disease. Breasts are not necessary for sexual satisfaction, and men and women have different psychological experiences, while the exaggerated expectation of “being too good at sex to be ideal for sex” is inevitable among the public. Couples should have realistic expectations of each other’s sexual performance based on their physical-mental health status. This model teaches couples how an unbalanced physical condition such as an illness or physiological changes following aging can affect the quality and quantity of sex and balance couples’ sexual expectations by creating cognition in individuals (McCarthy et al., 2008).

*Objective*

This study aimed to determine the effect of good enough sex model-based counseling intervention on the body image in women surviving breast cancer.

## Materials and Methods

*Patients and Methods*

This study is a quantitative randomized controlled clinical trial (RCT). The study aimed to determine the effect of couple-centered group counseling based on the GES model on sexual performance, satisfaction and relationship of couples surviving breast cancer. After registering the study in the Iranian Registry of clinical trials, the researcher started the sampling process from Omid hospital (Urmia, Iran). Initially, sampling was performed via convenience sampling. Participants were chosen from the population of married women with breast cancer referred to Omid hospital who met the inclusion criteria. The inclusion criteria for this study was: women of childbearing age (18-49 years), married, the presence of a spouse at least two weeks a month, non-pregnant and non- breastfeeding, definitive diagnosis of breast cancer (filing a case in Omid hospital in Urmia Requires written confirmation of the disease by an oncologist), negative medical history for chronic disease other than breast cancer, history of one of the mastectomy methods, stage I, II, III, at least six months from and a maximum of 5 years since the completion of radiotherapy and chemotherapy, no history of attending training classes or therapy and counseling sessions on sexual and reproductive health and no history of sexual dysfunction of the spouse. The exclusion criteria included: the occurrence of any mental or physical illness, trauma or accident for the patient or the patient’s spouse with a confirmed impact on sexual function, conception and pregnancy during the study, unwillingness to continue the study by the participant, refusal to attend the counseling sessions by the participant or the spouse for one session or more, participation in training or sexual counseling sessions outside the concept of the study..

Sample size was determined to be 42 people for each group and 50 people (100 people in total) were eventually enrolled by rounding the number and adding 20% loss for each group. Sampling in this study was done via convenience sampling. Hence, all eligible individuals entered the study after receiving a complete explanation of the purpose of the study and providing written consent. Next, the samples were divided into two groups of intervention and comparison by blocked randomization method. All possible modes were considered for placing the letters A and B in the four blocks, which came to a total of 6 modes. These 6 cases were numbered from 1 to 6 and the number of required 4-unit blocks was determined based on the number of samples studied. Since the sample size required for the study was 100 people, 25 blocks of 4 were formed. Then, according to the required number of blocks (25 blocks), random numbers were arranged in a row based on a table of random numbers; numbers greater than six were not considered. Then, based on the order of the numbers extracted from the table, the blocks corresponding to each number were listed in order. Finally, when the samples entered the study, each person took a specific letter in the order obtained. For example, according to the order (AABB / ABAB), the 5th person was placed in group A (intervention). Finally, the samples were divided into two groups of intervention and comparison based on the quadratic blocking method. At this stage, a questionnaire of demographic information and breast cancer status was completed by the researcher. Then, for the intervention group (group A), the designed intervention was performed while the subjects of the control group (group B), received educational content in the form of 4 one-hour voice files in the Telegram group chat (form of social media platform commonly accessible in Iran) at the end of the study. It should be noted that during the study, both groups were closely followed for possible interventions performed for them in the hospital, such as referral to a counselor, participation in training classes, etc. The intervention for group A consisted of at least 4 couples counseling sessions lasting at least 90 and at most 120 minutes according to the GES model once a week.

It should be mentioned that the presence of spouses was mandatory for the first and second counseling sessions; However, in the third and fourth sessions, and especially the third session, in which women with breast cancer were supposed to talk about their sexual concerns and problems, the spouses may or may not have been present at the patient’s discretion to protect the patient’s privacy. In our study, only 21 husbands were present at the counseling sessions. In addition, a secure telephone number was obtained from the participants so that they could be accessed. The researchers direct contact number was also provided to the participating women so that they could ask questions and receive answers in between sessions.

Finally, the status of participants in both groups A and B was assessed 2 and 3 months after the last intervention session, using standard questionnaires BIS by telephone interview. During the study, one couple from the intervention group (due to unwillingness to continue participation) and 2 couples from the control group (due to unwillingness to continue mutual participation and another due to recurrence of the disease) were excluded from the study. To analyze the findings of the quantitative part of the study, descriptive statistics was used to present the information in frequency distribution tables. Depending on the type of data, the Chi-square test was used to examine the groups in terms of demographic variables and clinical characteristics of the participants. Repeated measurement test was used to evaluate the performance and sexual satisfaction of individuals in both groups at different time points during the study. All statistical calculations were performed taking into account Intention to treat and significance level P <0.05 and using SPSS software version 18. Study flow chart is shown in Table 1.

The questionnaires used in the study include two questionnaires. The questionnaire of personal characteristics (10 questions) included age of patients, age of spouse, level of patient’s education, level of education of spouse, employment status of spouse and patient, adequacy of family income, type and duration of marriage, children number, the current method of contraception; and information about the disease status of the person (5 questions) including the duration of the disease, age of onset of the disease, type of surgery, type of adverse treatments and degree of disease. Body image scale (BIS) assessed anxiety about cancer-related body image (Hopwood et al., 2001). BIS contains 10 questions with 4-point Likert scale scoring from 0 (not at all) to 3 (too much). The minimum possible score is zero and the maximum is 30, and higher scores indicating a positive mental image. The validity and reliability of this questionnaire were evaluated by Hopwood et al in the population of cancer patients and the Cronbach’s alpha coefficient of this questionnaire was 0.93 (Hopwood et al., 2001). This questionnaire was used for the first time in the country, so for its validity and reliability, it was first translated and re-translated into Persian by a senior English language expert. Then, for the content validity, the opinions of the relevant faculty members and experts were used. During a pilot study, it was completed by 30 patients with breast cancer and entered into the software and its reliability was calculated. The reliability of the mental image questionnaire after cancer using the Cronbach’s alpha coefficient was 0.89.

*Ethical approval*

The study was reviewed and approved by the committee of Tehran University of Medical Sciences (IR.TUMS.FNM.REC.1396.4865). Registration of trial protocol has been approved in Iranian registry of clinical trials (IRCT20120609009975N8, https://en.irct.ir/trial/42030). Before entering the study, written consent was obtained from all patients and sufficient information about the objectives of the plan and how to implement it and review the consequences and benefits of the intervention for patients were described. This study was extracted from the doctoral dissertation of Samira Barjasteh in reproductive health (#9511151002).

## Results

**Figure 1:**
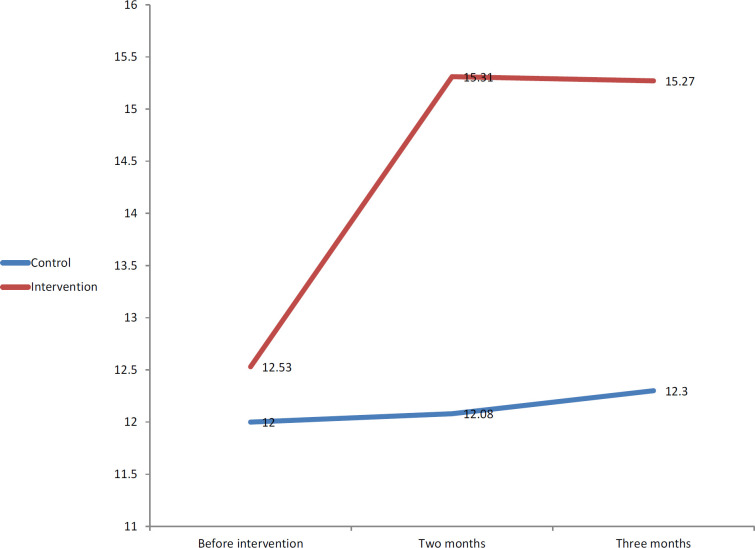
Comparison of Body Image Scale before Intervention, Two and Three Months after the Intervention in the Intervention and Control Groups

Table 2 and 3 compares the groups in terms of demographic and clinical characteristics of breast cancer survivors. In this study, the two groups were homogenous regarding age (P = 0.38), years since marriage (P = 0.6), number of children (P = 0.12), level of patient’s education (P = 0.41), level of spouse’s education (P = 0.71) ), economic status (P = 0.44), contraception method (P = 0.49), employment status of patients (P = 0.38), employment status of spouse (P = 0.52), duration of disease (P = 0.24), type of surgery (P = 0.23) and the disease stage (P = 0.22). Table 4 and Figure 1 present the mean body image scale in the intervention group at least at one time (before, 2 or 3 months after the intervention) which was statistically significant different from the others (P <0.001), while in the control group, there was no statistically significant difference between the mean body image scale at different times of the study (P = 0.26). Considering the significance of the ANOVA repeated measures test, the results of the Bonferroni test are shown in Table 4 to compares two different points in the study. According to Bonferroni test, it was observed that in the intervention group, the mean score of the body image scale of the body before the intervention was statistically significant difference compared to 2 months (P <0.001) and 3 months after the intervention (P <0.001). Thus, the mean score of the body image scale before the intervention in this group was less than the other two times. That is, the score of the body image scale in this group has increased after the intervention. However, there was no statistically significant difference between the time periods of two and three months after the intervention regarding the mean body image scale in the intervention group (P = 0.37). 

**Table 1:** Study Flow Chart in Women Surviving Breast Cancer

Session content	Specific objectives	
- Introduction and acquaintance of consultants and patients with each other - Explaining the objectives and rules of the meetings, asking for mutual respect, regular participation and etc. - Explaining the details of counseling sessions and the objectives of counseling and familiarize couples with the GES model - Explaining the importance of sexual activity, sexual education, definition of sexual satisfaction and performance and the factors affecting it, the effects of marital happiness on self, children, social relations and academic and professional performance, and relationship with spouse - Providing information about the reproductive cycle and effective methods of assisted reproduction and the mechanism of action of contraceptive methods and counseling on choosing the appropriate method - Explaining the four stages of the sexual response cycle with emphasis on the importance of recognizing the cycle of sexual "desire" - Explaining the effect of breast cancer and mastectomy on sexual function and its mechanism - Joint contract making technique to take note of needs and expectations in regards to sex, clarifying expectations, correcting misconceptions and superstitions about sex, and creating an optimistic attitude (counseling and teaching positive thinking style in interpreting sexual events and relationships) - Homework - Answering possible questions of couples - Summarize the first session of counseling and planning the next session	Implementation of the first three aspects of GES model - Explaining the importance of sex as a Crucial element of life - The importance of quality of sex and sexual satisfaction in the sexual development of couples Setting realistic expectations for sexual relations	1
- . A review and summary of the previous session - Nutritional recommendations, sleep hygiene, stretching and exercising and recommendations for daily walks for couples - Observing health tips and ensuring the selection of a suitable contraception method - Teaching meditation skills and training of mindfulness, body examination and conscious attention to different parts of the body, conscious movements and three-minute breathing exercises (directing air as a tool to direct attention to yourself and establish the mind in the moment) - Providing a timeline to record the thoughts and mental status of the person in instances of desirable and pleasant sexual experiences, unpleasant experiences, disgust, muscle contractions and emotional pressures - Teaching couples to enjoy each other (teaching lust and sexuality, stereotypes, misconceptions, fantasies and mental errors about sex) - Homework - . Answering possible questions of couples - Summarizing the second consultation session and planning the next session	Implementation of the fourth, fifth and sixth dimensions of GES models - Emphasis on physical health and healthy habits for reviving sexual health - Behavioral training and counseling (mindfulness) - Paying equal attention to gaining sexual pleasure as much as achieving proper sexual function	2
- . Considering the problems and concerns noted by the couple during the previous week - Changing expectations regarding the length of the period of sexual arousal, sexual vigor and orgasm - Benefiting from sexual fantasies - Advice on visual adaptation to anatomical changes - Helping to maintain verbal and emotional relationship with the spouse and emphasizing the importance of flirting and foreplay - Explaining the pattern of sexual changes and possible physical problems after treatment and focusing on the five goals in sex - Identify five principles of sex (1- Reproduction 2- Reducing stress and anxiety 3- Sexual pleasure 4- Self-esteem and trust 5- Intimacy and satisfaction with the relationship), Acceptance of multiple objectives by both parties, avoidance of one sided and inflexible objectives and clarification of the sex schedule - Explaining the different positions of intimacy and sexual arousal styles - Three styles of sexual arousal with a focus on body and interpersonal activities, sexual and emotional pleasure, daydreaming and role playing - Combining styles and creating variety in the relationship - Homework - . Answering possible questions of couples - Summarizing the third consultation session and planning the next session	Implementation of the seventh, eighth and ninth dimensions of the GES model - Counseling to overcome sexual fears and worries - Training to have flexible sexual experiences - Letting go of idealistic ideas about sex - Defining objectives in sex - Getting familiar with sexual arousal styles	3
- Counseling and education about the physical and psychological differences between men and women at each stage of the sexual cycle - Explaining the factors affecting sexual satisfaction and subsequent marital satisfaction - Communication skills training, skills to create and maintain intimacy (training to talk, listen empathetically, clarify hidden assumptions to resolve emotional sensitivities and share emotions in order to create intimacy) - Counseling and training in trust, mutual acceptance, freedom and deep appreciation of the relationship. Giving a special nature to sex such as cuddling or giving nicknames to sexy parts of the body and adding unique experiences in couples' relationships - . Answering possible questions of couples - Summarizing the whole process of references - Planning to follow-up the performance status and sexual satisfaction of the clients in 2 and 3 months after the fourth session	Implementation of the tenth, eleventh and twelfth dimensions of the GES model - Getting familiar with gender differences and acceptance of similarities and differences - Strengthening communication skills - Sexual desire cycle training and counseling to personalize sexual desire	4

**Table 2:** Comparison of the Demographic Characteristics of the Patients in the Intervention and Control Groups

Variable		Control group		Intervention group		Statistics
		%	N	%	N	
Age of patient (year)	18-28	18	9	12	6	X^2^=1.89
	29-38	36	13	38	19	P=0.38
	39049	56	28	50	25	
Marriage duration (year)	0.5-2	4	2	6	3	P=0.6
	Two-five	12	6	8	4	
	Five-ten	20	10	30	15	
	>10	64	32	56	28	
Number of children	0	46	23	34	17	X^2^=0.07
	1	30	15	50	25	P=0.12
	≥2	24	12	16	8	
Level of patient's education	Illiterate	10	5	14	7	X^2^=0.82
	Under diploma	54	27	40	20	P=0.41
	Diploma	20	10	32	16	
	Collegiate	16	8	14	7	
Level of spouse's education	Illiterate	28	14	24	12	P=0.71
	Under diploma	28	14	22	11	
	Diploma	40	20	46	23	
	Collegiate	4	2	8	4	
Economic status	Income more than expenses	16	8	24	12	X^2^=0.66
	Income less than expenses	18	9	22	11	P=0.44
	Income equals expenses	66	33	54	27	
Method of contraception	Condom	30	15	40	20	P=0.49
	Contraceptives	4	2	6	3	
	Discontinuous	30	15	24	12	
	Progesterone ampoules	4	2	2	1	
	IUD	30	5	20	10	
	Without prevention	2	1	8	4	
Occupation of patient	Employed	26	13	34	17	X^2^=0.76
	Housewife	74	37	66	33	P=0.38
Occupation of spouse	Unemployed	14	7	6	3	X^2^=3.18
	Labor	18	9	12	6	P=0.52
	Employed	22	11	28	14	
	Self-employed	34	17	36	8	
	Retired	12	6	18	9	
						

**Table 3:** Comparison of the Clinical Characteristics of the Patients in the Intervention and Control Groups

Variable		Control group		Intervention group		Statistics
		%	N	%	N	
Duration of disease (year)	1-5	38	19	26	13	X^2^=2.80
	5-7	34	17	50	25	P=0.24
	7-10	28	14	24	12	
Type of surgery	Mastectomy	82	41	72	36	X^2^=1.41
	Lumpectomy	18	9	28	14	P=0.23
Grade	I	22	11	20	10	X^2^=2.96
	II	40	20	56	28	P=0.22
	III	38	19	24	12	

**Table 4:** Comparison of Body Image Scale before Intervention, Two and Three Months after the Intervention in the Intervention and Control Groups

Body image scale	Control	Intervention	Statistics
	Mean± SD	Mean± SD	
Before intervention	12±3.95	12.53±3.83	F=1.66
Two months	12.08±4.03	15.31±3.50	P=0.001
Three months	12.30±4.05	15.27±3.48	
Tests	F=1.33	F=8.18	F=13.95
	P=0.26	P=0.001	P=0.001

## Discussion

The results of the demographic characteristics of the participants in the present study showed that the majority of women participating in the intervention and control group (50%) were aged between 39 to 49 years. Such a finding is consistent with the results of the study of Sharifian et al., (2015) which reported that the average age of onset in Iran is 50 years. Keeping in mind that the age-gender-pyramid of Iranian population is slightly younger compared with other countries, patients with a younger age at diagnosis account for a bigger proportion of all patients compared with patients with an older age at diagnosis (Sharifian et al., 2015). Couples with more than 10 years of married life (56%) had the highest frequency among the participants which is due to the higher proportion of participants in their third or fourth decade of life in this study. Also, in this study, condoms (40%) were the preferred method of contraception among the participants. This finding is in line with the reports made by Anderson et al., (2011) and Dominic et al., (2015) which showed that the majority of breast cancer survivors tend to use condoms due to lack of information or fear in regards to hormonal methods (Anderson et al., 2011; Dominic et al., 2015).

However, the results of a study by Madi et al., (2019) named the use of IUDs as the most common method of contraception by women surviving breast cancer. Considering that 61% of the participants in their study benefited from counseling sessions for fertility and choice of contraception, it seems logical to assume that this inclination towards the use of IUDs is influenced by education and counseling in their study community (Mody et al., 2019). Taking into account that the failure rate of condoms in the general population is 18%, a significant number compared with the less than 1% failure rates reported for IUDs, the inclination towards use of condoms for contraception increases the likelihood of unwanted pregnancies, introducing a new crisis into the already complicated course of breast cancer (Trussell et al., 2011). Also, in 50% of the participants, the duration of the disease in the present study was reported to be 5-7 years. This finding seems to be warranted when keeping in mind that women diagnosed in the third and fourth decade of their lives accounted for the highest proportion of the participants. Also, according to the findings of the study by Iligard et al, the first 5 years of survival are the most vital time period for breast cancer survivors in terms of the need for supportive and educational care (Trussell et al., 2011).

In this study, mastectomy with 70-80% frequency was identified as the most common type of surgery undergone by breast cancer survivors. The results of this study are in line with the results of Najafi et al which reported that mastectomy is the standard treatment in Iran (Najafi et al., 2015). 

Also, in examining the degree of disease, the research team with second-degree breast cancer patients had the highest number of participants. This finding is consistent with some studies is consistent with the studies of (Alizadeh et al., 2018; Tabrizi et al., 2017) and inconsistent with the results of the study of Minicozzi et al., (2019). As mentioned earlier, the reason for the discrepancy seems to be the differences in fields of research in the studies. Other studies conducted in Iran show the bitter fact that breast cancer detection and diagnosis in Iranian women often occurs in the advanced stages of the disease (Rafiemanesh et al., 2016). As an example in this regard Tabrizi et al gave us the dispiriting news that only 12% of women in the fourth and fifth decade of life had experience with mammography and there was a significant gap in breast self-examination or ultrasound studies in younger women (Tabrizi et al., 2018). In the present study, the intervention and control groups were homogeneous in terms of disease characteristics, personal and demographic information, and economic and social status (P˃0.05), as well as any other factors that could potentially influence the sexual function, quality of life and mental body image in women (Kowalczyk et al., 2019). In this regard, the results of a study conducted in Sari to investigate the effect of midwifery counseling support program on improving the body image of women surviving breast cancer showed that patients with breast cancer in the intervention group compared to the control group had better body image scores. Therefore, it seems that counseling interventions can inform patients about the course of the disease, its complications and, more importantly, practical strategies to improve self-concept (Hamzehgardeshi et al., 2017). This study, similar to the present study, was a group counseling of couples about improving the body image. But no follow-up was done for a long time and it was enough to check the condition of the participants immediately after the intervention.

Breast cancer treatment undeniably enhances a woman’s sexual function and fertility, her body integrity, and her perception of sexual self-concept. Education and counseling are needed to better understand how treatment affects the various aspects of women’s health, identity and relationships by health care providers (Saeedi et al., 2019).

The results of a review study by Male et al showed that the majority of women face many problems regarding sexual health and secondary self-concept following breast cancer, however, reports indicate a feeling of inadequate care or lack of counseling and treatment services in these areas are provided by health care providers. The results of this study were consistent with the present study to emphasize the effectiveness of GES model-based counseling (Male et al., 2016). Family support and increasing knowledge are of great factors to improve quality of life in patients with cancer especial to breast cancer (Kong and Guan, 2019; Ahmed et al., 2020; Falah et al., 2018; Zaker et al., 2019; Amarsheda et al., 2021; Vashistha et al., 2019). Kalaitzi et al., (2007) showed that five sessions of couple-centered sexual therapy focusing on improving interpersonal relationships and intimacy can improve their scores of depression and anxiety, as well as improve their body image and sexual satisfaction, which was consistent with the results of the present study. We found that couples counseling based on the GES model with emphasis on physical health and healthy behavioral habits, training and counseling on behavioral exercises and attention to sexual pleasure can promote a positive mental image of the body which is consistent with the study by Pintado et al in Mexico. In this study, mindfulness and self-awareness training (8 sessions) without follow-up was useful in improving the psychological and emotional changes related to perceived body image in patients surviving breast cancer (Pintado and Andrade, 2017). Due to the nature of the intervention implemented in this study, which concerned sexual issues of individuals, and keeping in mind the social and cultural prohibitions regarding this issue in Iran, individuals may have deep-seated internal red lines that prevent them from talking openly and honestly about marital problems and sexual dysfunction. The researcher tried to lessen this limitation by conducting the sessions in a private and appropriate setting and establishing a close and friendly interactions for consultation.

In conclusion, Good Enough Sex (GES) model-based sexual counseling intervention can significantly increase the body image of women surviving breast cancer. However, in order to improve the psycho-sexual condition of breast cancer patients, it is recommended that in addition to the interventions of the present study, psychosocial and multidisciplinary be performed in studies with larger sample size. 

*Key point*

The mean of body image in women surviving breast cancer after getting GES model-based sexual counseling intervention increase.

## Author Contribution Statement

FF, SB and ZK designed the study. SB, SRD, FF performed the experiments. SB, SRD collected data from patients and helped in performance of experiments. SB and SN prepared the primary draft after analysis. All authors read and signed the final paper.
